# Effect of *Smilax china* L.-containing serum on the expression of POLD1 mRNA in human hepatocarcinoma SMMC-7721 cells

**DOI:** 10.3892/etm.2013.1264

**Published:** 2013-08-19

**Authors:** BO CAO, ZIHAN ZHANG, YUQIN ZHANG, JIAQUAN LI, GANG LIANG, JIANGHONG LING

**Affiliations:** 1 Department of Traditional Chinese Medicine, The First Affiliated Hospital of Guangxi Medical University, Nanning, Guangxi 530021, P.R. China; 2 Experimental Center for Medical Sciences, Guangxi Medical University, Nanning, Guangxi 530021, P.R. China; 3 College of Basic Medicine, Guangxi Medical University, Nanning, Guangxi 530021, P.R. China

**Keywords:** *Smilax china* L., drug-containing serum, SMMC-7721 human hepatocarcinoma cell, POLD1

## Abstract

Bock greenbrier rhizome, also known as *Smilax china* L. rhizome, induces heat clearing and detoxification and dispels wind dampness. Additionally, this Chinese medicine has been shown to function as an anticancer compound in various types of cancer. The aim of the present study was to investigate the mechanism by which *Smilax china* L.-containing serum suppresses SMMC-7721 human hepatocellular carcinoma (HCC) cell growth as well as to determine its effect on the expression of DNA polymerase δ catalytic subunit gene 1 (POLD1). SMMC-7721 human HCC cells were cultured with serum containing various amounts of *Smilax china* L. for 24 h. The cells were also cultured in blank serum or serum containing a drug used in Western medicine (cyclophosphamide; CTX) as a positive control. HCC cell growth and proliferation were determined using the 3-(4,5-dimethylthiazol-2-yl)-2,5-diphenyltetrazolium bromide (MTT) assay. Cell cycle distribution and apoptosis were analyzed by flow cytometry, and the expression of POLD1 mRNA was detected by quantitative polymerase chain reaction (PCR). The number of cells following culture with *Smilax china* L.-containing serum was observed to be decreased. There was significant growth inhibition in the *Smilax china* L.-treated cells (shown in the high concentration serum group, volume fraction 30%), which was significantly different from the inhibition observed in the control group (P<0.05). Among the various cell cycle phases following culture, the percentage of cells in the S phase was significantly increased, and the percentage of cells in the G0/G1 phase was decreased; these percentages were significantly different from the percentages of the control cells (P<0.05). The results obtained following quantitative PCR showed a significant reduction in POLD1 expression. *Smilax china* L.-containing serum directly suppressed cell growth and induced the apoptosis of human HCC cells. However, the number of cells in the S phase was reduced. This mechanism is suggested to be associated with the suppression of POLD1 expression.

## Introduction

Bock greenbrier rhizome (1), also known as *Smilax china* L., induces heat clearing and detoxification and dispels wind dampness according to traditional Chinese medicine. It is used to treat pain associated with rheumatic arthritis and injuries from falls, fractures, contusions and strains in traditional Chinese medicine. There have been numerous studies showing that *Smilax china* L. has certain antitumor effects against various types of cancer, including lung, stomach, liver and colon cancer. The researchers screened 50 kinds of Chinese herbals by MTT method and they found out Smilax China L has antitumor effects (2). Compositions of Smilax China L inclusions are complex, including: steroidal saponins, flavonoids, polyphenols, stilbene type and tannins. Which have antitumor effects (3,4). Study found out apigenin have induced effect on C50 and 308 skin cells in mice and human leukemia HL 60 cell cycle arrest at G2 / M phase (5). However, the underlying mechanism of its antitumor activity remains to be elucidated (6). DNA polymerase delta δ of B family (DNA polymerase δ, pol δ) is the only one enzyme With the relevant to cell cycle, and plays an important role in DNA replication, repair, restructuring, and cell cycle regulation (7). POLD1 gene is the key of DNA polymerase delta catalytic subunit gene, and plays an important role on cell growth and differentiation. OU (8) detected POLD1 gene expression by RT-PCR, and found out that the expression in liver cancer tissue was significantly higher than tissue adjacent to carcinoma. The research showed that primary liver cancer associated with POLD1 gene. Basis on these studies, we have done deep research.

In the present study, serum pharmacological methods, 3-(4,5-dimethylthiazol-2-yl)-2,5-diphenyltetrazolium bromide (MTT) assay, flow cytometry and fluorescence quantitative polymerase chain reaction (PCR) were used to determine the effects of bock greenbrier rhizome on human hepatocellular carcinoma (HCC) cell growth and the expression of DNA polymerase δ catalytic subunit gene 1 (POLD1). The mechanism underlying the antitumor activity of *Smilax china* L. in HCC cells was also investigated.

## Materials and methods

### Drugs

*Smilax china* L. was purchased from the Guangxi Institute of National Medicine (Nanning, China). The *Smilax china* L. (200 g) was soaked in water for 30 min followed by gentle boiling for 30 min. The mixture was then cooled for 30 min and the residue was filtered to obtain the extract. This procedure was repeated twice, and the resulting products were mixed. The drug extracts were reduced to a volume of 100 ml, which contained 2 g/ml crude drug, and stored. Cyclophosphamide (CTX) was provided by Shanghai Fahrenheit Pharmaceutical Co., Ltd. (Shanghai, China).

### Animals and cell lines

Adult Sprague Dawley (SD) rats were purchased from the Experimental Animal Center of Guangxi Medical University (Nanning, China). The SMMC-7721 HCC cell line was obtained from the Cancer Institute of Guangxi Medical University (Nanning, China). The study was approved by the Ethicical Review Committee of The First Affiliated Hospital of Guangxi Medical University (Nanning, China).

### Animal model and treatment

Healthy SD rats (weight, 220–250 g) were allocated to the following three groups: blank control (group A; n=10), CTX (group B; n=10) and *Smilax china* L. (group C; n=10). Group A was given a gavage of saline, and group C received a gavage of *Smilax china* L. at a dose of 1.5 ml/100 g of body weight. The rats of group B were treated with an intramuscular injection of CTX at a dose of 25 mg/kg. According to the serum pharmacological flux method (9), the animals were dosed twice a day for 3 days. The animals were given water while fasting for 12 h. Blood was drawn following the last intramuscular injection or intra-gastric administration. Following treatment, blood samples were obtained from the abdominal aorta when the rats were under anesthesia with pentobarbital sodium (0.35 ml/100 g). All the animals were treated humanely according to the institutional guidelines of the Ethics Committee of The First Affiliated Hospital Of Guangxi Medical University. The blood samples were centrifuged at 1,006.2 x g for 15 min at 4°C to completely remove the cellular components. After incubating in a water bath at 56°C for 30 min (to achieve inactivation), the sample was sterile filtered through a 0.22-*μ*m microporous membrane filter. The supernatant was collected and stored at −20°C.

### Cell culture

All the cells were cultured in Roswell Park Memorial Institute (RPMI)-1640 (Thermo Fisher Scientific, West Palm Beach, FL, USA) supplemented with 10% fetal bovine serum (FBS; Beijing Solarbio Science & Technology Co., Ltd., Beijing, China) in a humidified incubator with 5% CO_2_ at 37°C. The culture solution was replaced every 1–2 days, and the cells were passaged once. Exponentially growing cells were selected for the experiments.

### MTT assay

Exponentially growing cells were harvested by trypsin digestion and made into single-cell suspension after cell counting. After adjusting the cell concentration to 1×10^4^/ml, the cells were added to a 96-well plate (100 *μ*l per well). The cells were supplemented with 10% FBS and stored in a humidified incubator with 5% CO_2_ at 37°C. After the cells had been cultured for 24 h, the culture medium was removed. The wells were divided into a blank control low concentration group (10 *μ*l blank rat serum + 90 *μ*l RPMI-1640 per well), a blank control high concentration group (30 *μ*l blank rat serum + 70 *μ*l RPMI-1640 per well), a CTX low concentration group (10 *μ*l blank rat serum + 90 *μ*l RPMI-1640 per well), a CTX high concentration group (30 *μ*l blank rat serum + 70 *μ*l RPMI-1640 per well), a *Smilax china* L. low concentration group (10 *μ*l blank rat serum + 90 *μ*l RPMI-1640 per well) and a *Smilax china* L. high concentration group (30 *μ*l blank rat serum + 70 *μ*l RPMI-1640 per well). After continuous culture for 24 h, 10 *μ*l MTT (Sigma, St. Louis, MO, USA) was added to each well. This mixture was allowed to incubate for 4 h. Subsequently, 100 *μ*l dimethylsulfoxide (DMSO) was added to each well. The plate was oscillated for 10 min until the MTT-formazan was completely dissolved. The absorbance was measured at 490 nm to determine the cell viability. Each experiment was performed three times.

### Apoptosis detected by flow cytometry

The exponentially grown SMMC-7721 cells were harvested (using a 2.5 g/ml trypsin digestion), made into a single cell suspension and counted. After the cell concentration was adjusted to 4×10^4^/ml, the cells were added to a 6-well plate (2 ml per well) supplemented with 10% FBS and grown in a humidified incubator with 5% CO_2_ at 37°C. After 24 h, the culture medium was removed. For the apoptosis analysis, the SMMC-7721 cells were plated into 6-well plates and treated with various quantities (10–30%) of blank serum, and serum containing either CTX and or *Smilax china* L. for 24 h. The cell culture fluid was then collected. The cells were washed once with phosphate-buffered saline (PBS) at 4°C, subjected to trypsin digestion (2.5 g/l) and then placed in cell nutrient solution. The cells were then blended and centrifuged at 447.2 × g for 5 min. Then, the supernatant was removed. The cells were incubated in the dark for 10 min at room temperature before adding 500 *μ*l binding buffer, 5 *μ*l Annexin V-fluorescein isothiocyanate (FITC) and 5 *μ*l propidium iodide (PI; Beijing DingGuo ChangSheng Biotechnology Co., Ltd., Beijing, China). The apoptotic rates were determined by flow cytometry (BD Biosciences, Franklin Lakes, NJ, USA). The percentage of early apoptotic cells was calculated through the determination of Annexin V-positive and PI-negative cells, and the percentage of late apoptotic cells was calculated through the determination of Annexin V-positive and PI-positive cells.

### Cell cycle detection by flow cytometry

The cells (plated at a density of 4×10^4^/ml per well in 6-well plates) were collected by centrifugation. The cells were washed twice with PBS at 4°C. Subsequently, the cells were fixed in 1 ml cold 70% ethanol and fixed at −20°C overnight. Centrifugation was repeated. The fixed cells were washed once with PBS at 4°C, treated with 0.2 ml PBS and RNase A (50 *μ*g/ml), and incubated for 30–45 min at 37°C after a 1-min ice bath. The cells were stained with PI (65 *μ*g/ml; Beijing Tripod Countries Biological Technology Co., Ltd.) in the dark for 30 min at 4°C. The cell cycle was analyzed by flow cytometry (BD Biosciences).

### Quantitative PCR

The total RNA was extracted using TRIzol solution (Invitrogen, Carlsbad, CA, USA), according to the manufacturer’s instructions. RNA was then converted to cDNA by reverse transcription. The reactions were placed in a 96-well plate using a preheated real-time instrument (ABI 7500 HT; Applied Biosystems, Foster City, CA, USA). The POLD1 specific primers were: 5′-GACTACACGGGA GCCACTGTCA-3′ (sense) and 5′-GTAACACAGGTTGTG GGCCATC-3′ (antisense), with an amplicon length of 117 bp. β-actin (Gene ID: 60) was used as an internal control for RNA integrity with the following specific primers: 5′-AACTCCATCATGAAGTGTGA-3′ (sense) and 5′-ACTCC TGCTTGCTGATCCAC-3′ (antisense) (length, 247 bp). Each 10 *μ*l reaction volume contained 1 *μ*l cDNA, which was synthesized as described above. The reaction also contained 10 *μ*l Power SYBR^®^-Green Master mix and 1 *μ*l each pair of the oligonucleotide primers described above. Each reaction was performed thrice. The cycling parameters were as follows: 95°C for 10 min, followed by 40 cycles of PCR amplification performed at 95°C for 15 sec, annealing at 62°C for 31 sec, and extension at 95°C for 15 sec. The Ct value was measured during the exponential amplification phase. The data were analyzed using the comparative threshold cycle (2^−ΔΔC_t_^) method.

### Statistical analysis

Data are expressed as the mean ± SEM. The differences among the groups were determined by ANOVA analysis, and comparisons between two groups were analyzed by SNK analysis. Differences between two groups were determined using Student’s t-test. P<0.05 was considered to indicate a statistically significant difference.

## Results

### Cell growth inhibition

The growth of SMMC-7721 human hepatoma cells was inhibited by bock greenbrier rhizome, as detected by MTT assay (Table I). Serum containing CTX or *Smilax china* L., particularly in high concentrations, was demonstrated to inhibit the proliferation of the SMMC-7721 human HCC cell line. The highest rate of growth inhibition was observed in the cells treated with CTX-containing serum. However, the growth inhibition of the cells treated with *Smilax china* L.-containing serum was not significantly different compared with that of the cells treated with CTX-containing serum.

### Cell cycle and apoptosis detection

**Figure 1.**
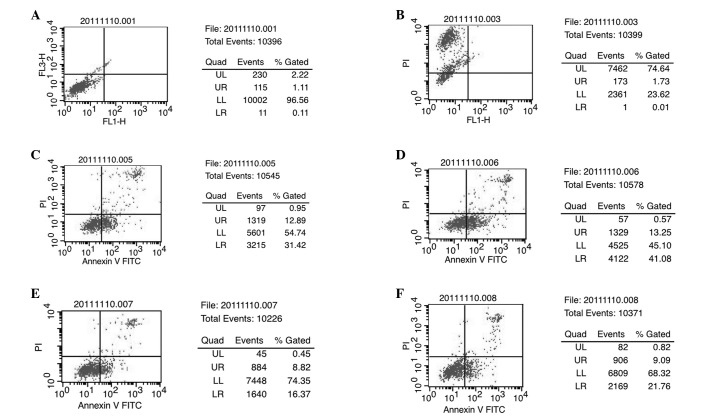
Percentage of apoptotic SMMC-7721 cells following treatment with serum from various groups. (A) Blank low-concentration group; (B) blank high-concentration group; (C) CTX low-concentration group; (D) CTX high-concentration group; (E) *Smilax china* L. low-concentration group; (F) *Smilax china* L. high-concentration group. CTX, cyclophosphamide.

**Figure 2.**
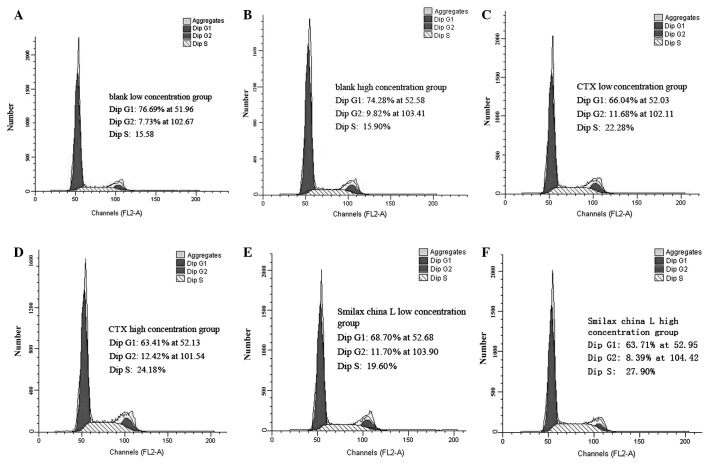
Cell cycle distribution of SMMC-7721 cells following treatment with serum from various groups. (A) Blank low concentration group; (B) blank high concentration group; (C) CTX low concentration group; (D) CTX high concentration group; (E) *Smilax china* L. low concentration group; (F) *Smilax china* L. high concentration group. CTX, cyclophosphamide.

Treatment with serum containing *Smilax china* L. or CTX inhibited the proliferation of SMMC-7721 cells (P<0.05). A particularly marked effect was observed with a high concentration of CTX-containing serum. The percentages of cells in the S phase increased following treatment with CTX- and *Smilax china* L.-containing serum (P<0.05). However, no significant differences were observed between the CTX- and *Smilax china* L. groups (Table II, Figs. 1 and 2).

### POLD1 mRNA expression

**Figure 3.**
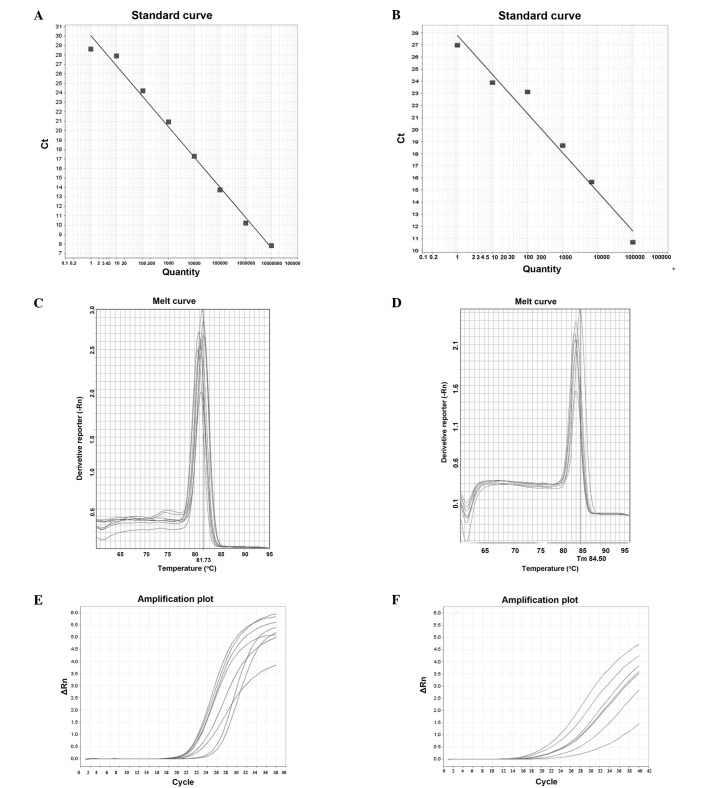
Diagrams of the reference genes used and the target genes investigated in quantitative polymerase chain reaction (PCR) analyses. (A) Internal control standard curve; (B) POLD standard curve; (C) internal control solubility curve; (D) POLD solubility curve; (E) internal control amplification curves; (F) POLD amplification curves compositions and multi-interference factors. POLD1, DNA polymerase δ catalytic subunit gene 1.

The mRNA expression of POLD1 was detected in HCC cells cultured with serum containing *Smilax china* L. The expression levels of POLD1 mRNA in the high and low concentration groups (0.45±0.1 and 0.28±0.06) were significantly different from the POLD1 mRNA expression levels in the blank group (P<0.05; Table III and Fig. 3).

## Discussion

HCC is one of the most frequent malignant tumors worldwide. Particularly, HCC is the fifth most common malignant tumor (10). Multiple gene interactions lead to primary HCC. Several relevant genes, including cancer (e.g., c-myc, c-fos, BC-12, cets2, Ras), tumor-suppressor (e.g., p16, p53, p21), cell cycle regulatory and cell apoptosis genes, as well as genes that maintain stability of the cell genome (11). The malignant proliferation of cancer cells is associated with the DNA bulk copy. DNA polymerase δ (pol δ) is the only DNA replication enzyme that is related to the cell cycle and plays a prominent role in DNA replication (12). The POLD1 gene is a key gene for the DNA polymerase δ catalytic subunit and plays an important role in cell growth and differentiation. Recent studies have shown that genetic mutations in the POLD1 gene are related to lymphoma, liver (8), colon and gastric cancer (13,14).

Serum pharmacology (15) uses medicated serum as an object of research. Serum pharmacology not only eliminates the interference of the physical and chemical properties of traditional Chinese medicine preparations, but it also reflects the last process of digesting and absorbing Chinese traditional medicine in the gastrointestinal tract. Moreover, serum pharmacology further reflects the biological transformation and production of a biological effect, and represents the real effective function of drug components that affect the body. Therefore, it is appropriate for Chinese medicine, which has complex chemical components.

In a previous study conducted by our group (16), HCC cells were cultured *in vitro* with serum containing 10 different concentrations of a Chinese medicine. MTT assay was then used to determine whether the medicated serum affected cancer cell proliferation. According to the results obtained, the growth of HCC cells was inhibited following *Smilax china* L. and CTX treatment. Based on these previous findings, a more detailed study was then conducted by our group.

*Smilax china* L. is an herb with a complex chemical composition and is classified as a Chinese herbal medicine. It has a complex set of ingredients including steroid saponins, flavonoids, polyphenols, stilbenes and tannins. *Smilax china* L. is known to expel wind and dampness as well as to scatter detoxification stasis (17,18). Previous studies have shown that *Smilax china* L. has an inhibitory effect on cancer cell growth (14,19). However, its mechanism remains unknown and further study is needed.

In the present study, we investigated the antineoplastic mechanism by which *Smilax china* L. affects SMMC-7721 cells using serum pharmacological methods. Tumor pathogenesis has a close association with suppression of apoptosis. In malignant tumor tissues, there are abnormal increases in the proliferation of malignant cells and marked reductions in cell apoptosis. Apoptosis defects constitute one of the pathogenic mechanisms of tumors. Therefore, the induction of apoptosis has the potential to inhibit and block tumor occurrence and development, thereby increasing the ratio of cell apoptosis to proliferation.

Apoptosis is a complicated cell death network and involves many components. Numerous studies have shown that one of the antineoplastic mechanisms of traditional Chinese medicine is the induction of apoptosis (20,21). In the present study, the serum of rats treated with high and low concentrations of *Smilax china* L. induced the inhibition of HCC cell proliferation and apoptosis *in vitro*. Thus, *Smilax china* L.-containing serum induced apoptosis, which inhibited SMMC-7721 cell proliferation *in vitro*.

The results obtained following cell cycle analysis showed that the number of cells in the S+G2/M phase increased following treatment with the serum from the *Smilax china* L.-treated rats (the number of cells in the S phase was significant) compared with the control cells. However, the number of cells in the G0/G1 phase decreased. Therefore, *Smilax china* L. is suggested to effectively mobilize cells from the G0/G1 to the S phase. Statistical analysis also indicated that the numbers of cells in the S+G2/M phase observed in the *Smilax china* L.-treated groups were not significantly different from those in the CTX-treated groups. Therefore, *Smilax china* L. is suggested to be a cell cycle non-specific drug, similar to CTX. *Smilax china* L. interferes with DNA and RNA function, particularly DNA function (22,23). *Smilax china* L. crosslinks with DNA and inhibits DNA synthesis. The effect on the number of cells in the S phase was the most evident. A study has confirmed that POLD gene expression is strictly regulated by the cell cycle (13). In the G1/S phase, or even in the early S phase, pol δ synthesis and POLD1 promoter activity increases, suggesting that POLD1 gene regulation occurs mainly in the G1/S phase.

Fluorescent quantitative PCR results showed that a clear downregulation of POLD1 mRNA expression was observed in the *Smilax china* L. high concentration group. The regulation of POLD1 mRNA expression by the tumor suppressors p53 and Sp1 through competing for the POLD1 promoter binding site is suggested to be a potential mechanism underlying the activity of *Smilax china* L. (24). It has been reported that p53 binds directly to the POLD1 promoter in the cell (25). Another potential mechanism is p21 (a p53 transcription product) binding. p21 restrains E2F1 release through binding to the POLD1 promoter at the E2F1 binding site, thereby inhibiting POLD1 activity (19). Moreover, p21 binds to proliferation cell antigen (PCNA), thus affecting the binding of PCNA and pol δ and hindering DNA replication (25). Furthermore, cyclin E/CDK2 indirectly regulates POLD1 promoter activity through the regulation of the downstream E2F1 family, which influences the expression of POLD1 mRNA (26). Song *et al* (27) demonstrated that the upregulation of POLD1 mRNA expression by CDE/CHR gene mutations is directly or indirectly regulated by p21 and E2F1. The regulation of POLD and the associations among p53, p21, CDE/CDK, the cyclin/CDK complex and POLD1 requires further study.

In conclusion, *Smilax china* L.-containing serum inhibits HCC cell proliferation and induces apoptosis. *Smilax china* L. also causes S phase cell cycle arrest in HCC cells. This mechanism is suggested to be associated with the inhibition of POLD1 gene expression.

## Figures and Tables

**Table I.** Effect of *Smilax china* L.-containing serum on the proliferation of human HCC SMMC-7721 cells (mean ± SEM, n=6).

Group	MTT (OD value)	Inhibition ratio (%)
Blank		
Low concentration	0.832±0.014	-
High concentration	0.722±0.011	-
CTX		
Low concentration	0.774±0.017	6.97^a^
High concentration	0.563±0.019	22.02^b^
*Smilax china* L.		
Low concentration	0.807±0.022	3^a^
High concentration	0.588±0.017	18.56^b^

a P<0.01 compared with blank low concentration group;

b P<0.01 compared with blank high concentration group. HCC, hepatocellular carcinoma; CTX, cyclophosphamide.

**Table II.** Effect of serum from each group on the apoptosis and cell cycle distribution of SMMC-7721 human HCC cells (mean ± SEM, n=3).

Group	Apoptosis ratio (%)	Cell cycle (%)		
		G0/G1	S	G2/M
Blank				
Low concentration	1.22±0.31	76.69±1.63	15.58±1.82	7.73±0.19
High concentration	1.74±0.27	74.28±1.42	15.9±1.2	9.82±0.22
CTX				
Low concentration	44.31±2.14^a^	66.04±1.53	22.28±1.3^a^	11.68±0.23
High concentration	54.33±3.43^b^	63.41±1.21	24.18±1.3^b^	12.41±0.09
*Smilax china* L.				
Low concentration	25.19±2.2^a,c^	68.7±1.8	19.6±2.1^a^	11.7±0.3
High concentration	30.85±3.1^b,d^	63.71±3.1	27.9±2.7^b^	8.39±0.4

a P<0.05 compared with blank low concentration group;

b P<0.05 compared with blank high concentration group;

c P<0.05 compared with CTX low concentration group;

d P<0.05 compared with CTX low concentration group. HCC, hepatocellular carcinoma; CTX, cyclophosphamide.

**Table III.** Expression of POLD1 mRNA in each group of SMMC-7721 human HCC cells (mean ± SEM).

Group	Ct value		2^−ΔΔC_t_^
	POLD1	β-actin	
Blank	26.53±0.21	25.08±0.17	1
*Smilax china* L. high concentration	26.70±0.11	24.47±0.19	0.45±0.1^a^
*Smilax china* L. low concentration	28.42±0.32	25.87±0.29	0.28±0.06^a^

a P<0.05 compared with the blank group. POLD1, DNA polymerase δ catalytic subunit gene 1; HCC, hepatocellular carcinoma.
